# Development and Validation of an Owner-Assessed Feline Acute Pain Scale: Validation and Agreement with Veterinary Scales

**DOI:** 10.3390/ani15192801

**Published:** 2025-09-25

**Authors:** Samolwan Rojsiripornchai, Sirirat Niyom, Nattika Koatsang, Sakunrat Kathatip, Teerapat Thunpattranon, Wutti Bunjerdsuwan, Tassanee Jaroensong, Suwicha Kasemsuwan

**Affiliations:** 1 Kasetsart University Veterinary Teaching Hospital, Faculty of Veterinary Medicine, Kasetsart University, Bangkok 10900, Thailand; r.samolwan@gmail.com (S.R.); nattika.k@ku.th (N.K.); sakunrat.kha@ku.th (S.K.); teerapat.thunp@ku.th (T.T.); wutti.b@ku.th (W.B.); 2 Department of Companion Animal Clinical Sciences, Faculty of Veterinary Medicine, Kasetsart University, Bangkok 10900, Thailand; tassanee.j@ku.th; 3 Department of Veterinary Public Health, Faculty of Veterinary Medicine, Kasetsart University, Nakorn Pathom 73140, Thailand; suwicha.k@ku.th

**Keywords:** acute pain, feline, internal consistency reliability, owner, pain scale, validity

## Abstract

Pain assessment in cats is challenging, especially for non-veterinarians. Most validated acute pain scales are designed for clinical use, limiting their applicability for pet owners. This study developed a feline acute pain assessment scale for owners and evaluated its criterion validity, internal consistency reliability, and agreement with three veterinary scales: Glasgow Composite Measure Pain Scale-Feline (CMPS-Feline), Feline Grimace Scale (FGS), and Colorado State University Feline Acute Pain Scale (CSU-FPS). Of 146 enrolled cats, 130 were analyzed after exclusions. The owner-assessed scale showed strong correlation with CMPS-Feline (rho = 0.66) and moderate correlations with FGS (rho = 0.53) and CSU-FPS (rho = 0.57) (all *p* < 0.001). Agreement was substantial with CMPS-Feline (kappa = 0.74), moderate with FGS (kappa = 0.44), and fair with CSU-FPS (kappa = 0.28) (all *p* < 0.001). Internal consistency was acceptable (Cronbach’s alpha = 0.76). Receiver operating characteristic analysis demonstrated good discriminatory ability for identifying cats requiring analgesia, with area under the curve values of 0.87 (CMPS-Feline), 0.79 (FGS), and 0.75 (CSU-FPS). A cut-off score of 9 achieved 96% sensitivity and 78% specificity relative to CMPS-Feline. These results support the scale’s potential as a valid tool for pain detection by cat owners in non-clinical settings.

## Simple Summary

Recognizing pain in cats can be difficult, particularly for people without veterinary training. Most existing pain assessment tools are designed for professionals and may be too complex for owners. This study developed a new acute pain scoring tool specifically for cat owners to help them better recognize when their cats are experiencing pain. The tool was tested in 146 cats, with 130 included in the final analysis, and compared with three veterinary pain scales: the Glasgow Composite Measure Pain Scale-Feline (CMPS-Feline), the Feline Grimace Scale (FGS), and the Colorado State University Feline Acute Pain Scale (CSU-FPS). The results showed that owner-assigned scores aligned well with veterinary assessments. A score of 9 or higher on this new scale was found to be the best point for identifying cats likely requiring analgesic treatment. This new tool could help pet owners recognize signs of pain earlier, so they can take their cats to the veterinarian sooner. Early pain detection can improve a cat’s well-being and lead to quicker, more effective care.

## 1. Introduction

Pain is a common condition that significantly compromises the quality of life in small animals [1,2,3,4]. However, since animals are non-verbal and unable to self-report, detecting pain can be inherently difficult. Recognizing pain in animals largely depends on the observation of behavioral signs and an understanding of potential causes to guide appropriate management by veterinarians and caregivers [5]. Early detection and accurate assessment are essential to ensure timely treatment, minimizing suffering and reducing the risk of maladaptive mechanisms that can lead to chronic or difficult-to-treat pain states [5]. As owners are often the first to notice behavioral changes in their pets, their ability to recognize and interpret these signs is crucial [1,6]. Nevertheless, owners may be uncertain whether the observed signs truly indicate pain and its severity, which can be problematic when deciding whether veterinary attention is needed and when communicating observations to veterinarians [6].

Pain assessment in cats remains particularly challenging due to their species-specific behavioral traits and their often subtle or ambiguous pain expressions [1,6,7,8]. To address these challenges, numerous studies have focused on identifying and validating indicators of feline pain, with a particular emphasis on behavioral expressions [7,9,10,11,12,13], as behaviors are widely recognized as reliable indicators of pain in small animals [4]. Examples in cats include hunched posture, altered mobility, social withdrawal, and changes in temperament or facial expression [1,5,6,13]. Acknowledging the clinical relevance of these behavioral signs, several specialized feline pain assessment tools have been developed, such as the UNESP-Botucatu Multidimensional Composite Pain Scale (UNESP-Botucatu MCP) [11], the Glasgow Composite Measure Pain Scale-Feline (CMPS-Feline) [10], the Feline Grimace Scale (FGS) [14,15], and the Colorado State University Feline Acute Pain Scale (CSU-FPS) [16].

Briefly, the CMPS-Feline assesses pain through behavioral observation, response to interaction, vocalization, response to palpation, and facial expression [10]. The CSU-FPS evaluates multiple behavioral parameters such as posture, activity, attitude, vocalization, and response to palpation [16]. The UNESP-Botucatu MCP incorporates multiple dimensions, including behavioral changes, physiological parameters, and response to palpation, to provide a comprehensive evaluation of pain in clinical settings [11]. The FGS, another tool widely used, evaluates pain based on five facial action units: ear, eye, whisker, muzzle, and head position [14,15].

Despite the availability of several feline pain assessment tools for clinical use, these instruments are primarily intended for veterinary professionals and may require training for accurate implementation [6]. Consequently, their utility outside of clinical settings—particularly in the home environment—is limited. Given that cat owners play a vital role in recognizing and managing pain [1], accessible and reliable tools for non-professional use are essential. However, few studies have focused on supporting owners in this task, and standardized tools specifically designed for non-professional use remain scarce [6,17,18,19,20]. As a result, owners may still struggle to identify pain in their cats. The development of a validated pain assessment tool for use by cat owners could facilitate early recognition and prompt intervention, ultimately improving feline welfare. Moreover, a tool that can be used in the home environment may minimize confounding stress responses triggered by hospital visits [21], thereby improving the accuracy of pain recognition and assessment.

The aim of the present study was to develop and validate an owner-assessed feline acute pain scale specifically designed to assist cat owners in evaluating acute pain in their pets. Specifically, the study evaluated the scale’s psychometric properties, encompassing its internal consistency reliability, diagnostic discrimination, and, importantly, its criterion validity through assessment of correlation and agreement with established veterinary pain assessment tools (the CMPS-Feline, FGS, and CSU-FPS). It was hypothesized that the scale would serve as a validated, practical tool for owners, demonstrating acceptable internal consistency, reliability, and validity, with assessments correlating with those obtained from established veterinary pain scales.

## 2. Materials and Methods

### 2.1. Animals

This study was approved by the Institutional Animal Care and Use Committee of Kasetsart University (Approval No. ACKU67-VET-003) for procedures involving animals, and by the Kasetsart University Research Ethics Committee for procedures involving human participants (Approval No. COE67/047). Written informed consent was obtained from all participating cat owners prior to their enrollment in the study.

This prospective study was conducted at Kasetsart University Veterinary Teaching Hospital during 2024 and 2025. A total of 146 cats and their owners were enrolled in the study. The inclusion criteria were as follows: cats of any age that were clinically stable and presented to the surgical examination room either for preoperative consultations or for postoperative follow-up examinations; those in unstable or severely compromised condition were not recruited. Participating owners were required to be the cat’s primary caregivers who were familiar with the animal’s usual behavior and daily routine. Individuals with minimal interaction with the cat, such as relatives who only accompanied the cat to the hospital, were not eligible. The exclusion criteria included the following: (1) healthy cats presented for elective procedures such as ovariohysterectomy, castration, or dental scaling; (2) cats with anatomical features that could impair accurate pain assessment based on facial expressions (the FGS and the CMPS-Feline), such as folded ear pinnae, or those that had undergone total ear canal ablation (TECA) or pinnectomy; (3) cats in a comatose state; (4) cases in which the veterinary pain assessment was disrupted or influenced by environmental factors, such as sudden loud noises (e.g., barking dogs) or someone unintentionally entered the room; (5) preoperative cats that had received analgesic medications prior to the veterinary pain assessment; and (6) cases with incomplete owner-reported data. Exclusion criteria were applied after initial enrollment and prior to final analysis.

The sample size was determined using G*Power software (version 3.1.9.7; Heinrich-Heine-Universität Düsseldorf, Düsseldorf, Germany) [22]. Assuming a correlation coefficient of 0.3 between the owner-assessed scale and veterinary pain assessment tools, with a significance level of 0.05 (two-tailed) and a statistical power of 90%, the minimum sample size was calculated to be 112 cats. To account for potential missing data and exclusions, approximately 25% more cats and owners were recruited. To ensure a representative range of pain levels for validating the owner-assessed pain scale, cats were recruited from three clinical groups expected to exhibit varying degrees of pain: (1) cats requiring orthopedic surgery (fracture or luxation repair), (2) cats requiring soft tissue surgical intervention, and (3) cats presented for postoperative follow-up examinations between 3 days and 2 weeks after surgery.

### 2.2. Study Design

#### 2.2.1. Development of the Owner-Assessed Feline Acute Pain Scale

The initial version of the owner-assessed feline acute pain scale was developed based on a comprehensive review of existing guidelines and educational materials from veterinary associations and academic institutions [1,5,23]. Behaviors and signs indicative of pain identified from these sources were subsequently evaluated and discussed by a panel comprising a board-certified veterinary anesthesiologist, a veterinary surgeon, and a feline medicine specialist. This expert consultation focused on selecting behaviors that could be reliably observed by owners in a home setting, thereby forming the foundation of the initial pain scale.

Content validity was further evaluated by one additional board-certified veterinary anesthesiologist and two experienced veterinary anesthetists, resulting in a Content Validity Index (CVI) of 1.0. Preliminary testing was performed on 23 cats, and feedback from participating cat owners was used to revise the owner-assessed pain scale, resulting in a finalized version with improved clarity and comprehensibility.

#### 2.2.2. Study Protocol

Each participating owner received a finalized questionnaire consisting of three sections. The first section collected demographic and relational information, including the cat’s and owner’s age, gender, and the perceived relationship between the cat and owner. Owners were asked to indicate the strength of this relationship by marking a point on a 100 mm horizontal line (owner–cat relationship score), where 0 represented “no relationship” and 100 indicated “the closest relationship imaginable”.

The second section consisted of the owner-assessed pain scale, which included six items rated on a three-point Likert scale and seven binary (yes/no) items. The six Likert-scale items evaluated signs such as: (1) hiding or decreased interest in play or social interaction; (2) poor coat condition or reduced grooming; (3) narrowed and dull eyes; (4) hunched posture or tucking of the tail or limbs under the body; (5) frequent shifting or restlessness during sleep; and (6) increased aggression. Owners were instructed to rate each behavior as “not present” (0 points), “sometimes observed” (1 point), or “clearly present” (2 points). The seven binary items addressed whether the cat: (1) reacted aggressively or defensively when touched in certain areas; (2) refused to be stroked or groomed; (3) spent more time sleeping; (4) slept in unusual or hidden places; (5) moved differently (e.g., limping, stiffness, or reluctance to jump); (6) showed changes in eating behavior (e.g., refusal to eat or increased selectivity); and (7) exhibited altered urination or defecation patterns (e.g., elimination outside the litter box). A “yes” response was scored as 3 points, and “no” as 0 points. The total score for the owner-assessed scale ranged from 0 to 33.

The third section included a visual analog scale (VAS) for pain assessment, represented by a 100 mm horizontal line. Owners were instructed to mark the point that best represented their perception of their cat’s current pain level, with 0 mm indicating “no pain” and 100 mm representing “the worst pain imaginable”.

On the study day, owners completed the pain scale independently once at the hospital. If owners encountered difficulties, a researcher assisted by reading or explaining the items. This design was chosen to provide a controlled setting to evaluate whether owners could understand and complete the scale independently, and to determine the extent to which assistance was required before considering its application in home-based settings. At the time of scoring, the cats had been transferred to a designated study room for veterinary assessment and were, therefore, not physically with their owners. Each cat was then evaluated by a veterinary anesthesiologist using the CMPS-Feline, the FGS, and the CSU-FPS. This veterinarian was blinded to both the cats’ medical records and the owner-assessed scores and evaluated all cats in the study to ensure consistency. All evaluations were conducted in a quiet, stress-free environment to minimize external influences. If a cat’s score met or exceeded the established thresholds for analgesic intervention (CMPS-Feline ≥ 5/20 [10], FGS ≥ 4/10 [24], or CSU-FPS ≥ 2/4 [16]), appropriate analgesic treatment was administered according to the hospital’s standard protocol.

#### 2.2.3. Statistical Analysis

All statistical analyses were performed using R software (version 4.4.1; R Foundation for Statistical Computing, Vienna, Austria). Descriptive statistics were used to summarize the data. Continuous variables were expressed as the mean ± standard deviation when normally distributed, and as the median with interquartile range (IQR) when not normally distributed. The normality of the data was assessed using the Shapiro–Wilk test. Differences in pain scores between the orthopedic, soft tissue, and postoperative groups were evaluated using the Kruskal–Wallis test, followed by Dunn’s post hoc test with Bonferroni correction for multiple comparisons. Criterion validity, which assesses the correlation between the owner-assessed pain scale and the established veterinary pain scales, was analyzed using Spearman’s rank correlation coefficient. Cohen’s kappa was calculated to evaluate the level of agreement between the owner and veterinary assessments. The internal consistency reliability of the owner-assessed feline pain scale was assessed using Cronbach’s alpha coefficient. Receiver operating characteristic (ROC) curve analysis was conducted to determine the sensitivity and specificity of the owner-assessed pain scale in identifying cats requiring analgesic intervention, and the optimal cut-off score was determined using the Youden index [25]. In addition, Spearman’s rank correlation was applied to evaluate associations between the owner-assessed pain scores and owner-related variables, including the VAS pain score reported by owners, the owner–cat relationship score, and owner age. Associations between categorical variables (e.g., owner gender) and owner-assessed pain scores were evaluated using the Chi-squared test. A *p*-value of <0.05 was considered statistically significant.

## 3. Results

### 3.1. Study Population

**Figure 1:**
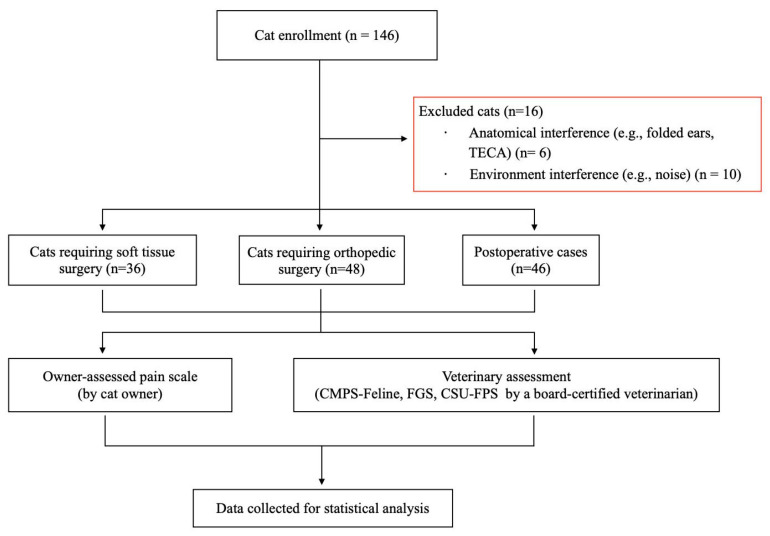
Flow chart of cat enrollment, exclusion criteria, and pain assessment methods.

Data from 130 cats and their owners were included in the final analysis (Supplementary Table S1). Sixteen cats were excluded: six due to anatomical features (five with folded ears and one that had undergone TECA), and ten due to excessive environmental stimulation during veterinary pain assessment (Figure 1).

Owners’ ages ranged from 13 to 84 years, with a median age of 34.5 years (IQR: 28.3, 45.0); the sample comprised 33 males and 97 females. The animal population included 48 cats in the orthopedic group (44 with fractures and 4 with joint luxations), 36 cats in the soft tissue group (8 with traumatic wounds, 5 with mammary gland tumors, 9 with skin tumors, 6 with visceral trauma, 5 with urolithiasis, 2 with atresia ani, and 1 with peritoneopericardial diaphragmatic hernia), and 46 cats in the postoperative group. The postoperative cases included 8 cats recovering from fracture repair, 15 from laparotomy, 6 from castration, 2 from peritoneopericardial diaphragmatic hernia repair, 2 from fistula tract excision, 2 from limb amputation, 4 from enucleation, 2 from penile amputation, and one each from bilateral mastectomy, chest drain placement, skin mass removal, esophageal stricture correction, and atresia ani repair. No cat was included in more than one group.

The breed distribution was as follows: 101 Domestic Shorthair, 10 Persian, 10 Scottish Fold, 3 American Shorthair, 4 British Longhair, 1 Maine Coon, and 1 Devon Rex. Of these, 71 were male and 59 were female cats. The demographic characteristics of cats in this study are presented in Table 1.

### 3.2. Pain Scores Across Clinical Groups

Median and IQR values of pain scores for each group, as assessed by the different pain assessment tools, are presented in Table 2. The orthopedic group exhibited significantly higher pain levels compared to the postoperative group across all veterinary scales and the owner-assessed scale, and significantly higher pain levels than the soft tissue group, as assessed by the CMPS-Feline and CSU-FPS. No significant differences were observed between the soft tissue and postoperative groups across all assessment tools.

### 3.3. Correlation, Agreement, and Internal Consistency

The owner-assessed feline pain scale demonstrated strong positive correlation with the CMPS-Feline (rho = 0.66, 95% confidence interval (CI): 0.50–0.72, *p* < 0.001), and moderate positive correlations with the FGS (rho = 0.53, 95% CI: 0.38–0.66, *p* < 0.001) and the CSU-FPS (rho = 0.57, 95% CI: 0.44–0.68, *p* < 0.001). Among the veterinary assessment scales, strong positive correlations were observed between the CMPS-Feline and the FGS (rho = 0.79, 95% CI: 0.70–0.85, *p* < 0.001), and between the FGS and the CSU-FPS (rho = 0.63, 95% CI: 0.51–0.73, *p* < 0.001). A very strong correlation was found between the CMPS-Feline and the CSU-FPS (rho = 0.83, 95% CI: 0.76–0.89, *p* < 0.001) (Table 3).

Substantial agreement was observed between the owner-assessed pain scale and the CMPS-Feline (kappa = 0.74, 95% CI: 0.57–0.91, *p* < 0.001). Moderate agreement was found with the FGS (kappa = 0.44, 95% CI: 0.23–0.59, *p* < 0.001), and fair agreement with the CSU-FPS (kappa = 0.28, 95% CI: 0.16–0.41, *p* < 0.001). Among the veterinary scales, moderate agreement was observed between the CMPS-Feline and the FGS (kappa = 0.57, 95% CI: 0.43–0.71, *p* < 0.001), while fair agreement was noted between the CMPS-Feline and the CSU-FPS (kappa = 0.38, 95% CI: 0.22–0.54, *p* < 0.001), and between the FGS and the CSU-FPS (kappa = 0.37, 95% CI: 0.19–0.56, *p* < 0.001) (Table 4).

The internal consistency of the owner-assessed feline pain scale was evaluated using standardized Cronbach’s alpha, yielding a coefficient of 0.76, which indicates good internal consistency. Interpretation of the coefficient is as follows: alpha < 0.65 = unsatisfactory; 0.65–0.69 = fair; 0.7–0.74 = moderate; 0.75–0.79 = good; >0.8 = excellent [27]. The recalculated alpha coefficients, based on deleting individual items, ranged from approximately 0.72 to 0.76.

### 3.4. Owner-Related Factors Associated with Pain Scores

The VAS pain score reported by owners was significantly correlated with the total score from the owner-assessed pain scale (rho = 0.62, 95% CI: 0.48–0.72, *p* < 0.001). In contrast, the owner–cat relationship score (median = 100 mm, IQR: 84.3, 100) and owner age (median = 34.5 years, IQR: 28.3, 45.0) were not significantly correlated with the owner-assessed scores (rho = −0.15, 95% CI: −0.32–0.05, *p* = 0.09 and rho = −0.04, 95% CI: −0.21–0.16, *p* = 0.691, respectively). Owner gender was also not associated with the owner-assessed pain score (*p* = 0.482), as determined by the Chi-squared test. The median score assigned by male owners was 9.0 (IQR: 7.0, 16.0), and by female owners was 9.0 (IQR: 5.0, 13.0). The majority of owners were able to complete the pain scale independently. However, a small number of owners (n = 5), all of whom were elderly (60, 72, 76, 78, and 84 years), required assistance from a researcher to complete the questionnaire.

### 3.5. ROC Curve Analysis

**Figure 2:**
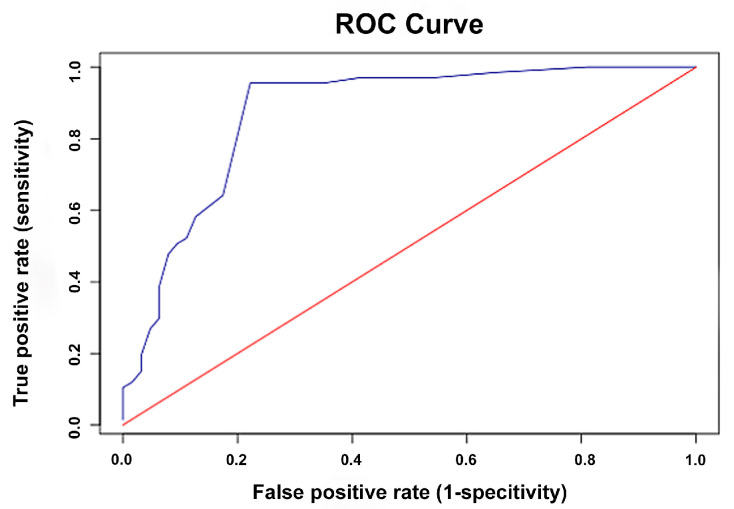
Receiver operating characteristic (ROC) curve for the owner-assessed feline pain scale in detecting pain requiring intervention as determined by the Glasgow Composite Measure Pain Scale-Feline (CMPS-Feline). The blue line represents the ROC curve for the owner-assessed feline pain scale, and the red diagonal line represents the line of no discrimination.

**Figure 3:**
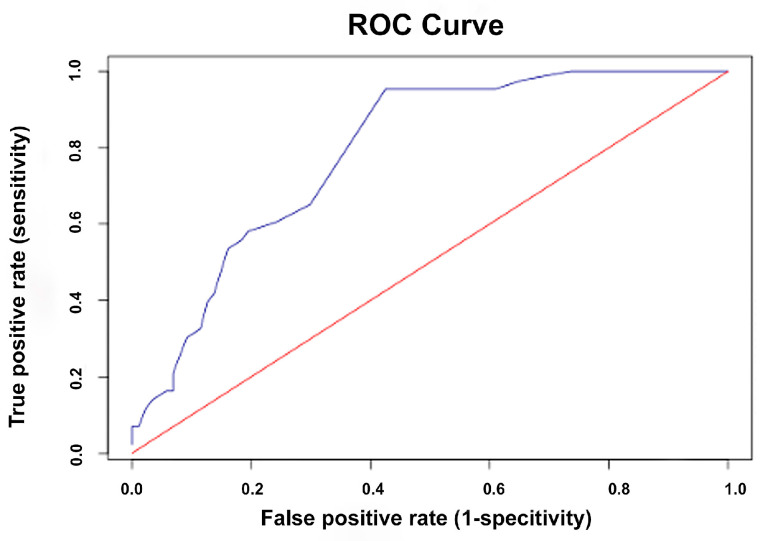
Receiver operating characteristic (ROC) curve for the owner-assessed feline pain scale in detecting pain requiring intervention as determined by the Feline Grimace Scale (FGS). The blue line represents the ROC curve for the owner-assessed feline pain scale, and the red diagonal line represents the line of no discrimination.

**Figure 4:**
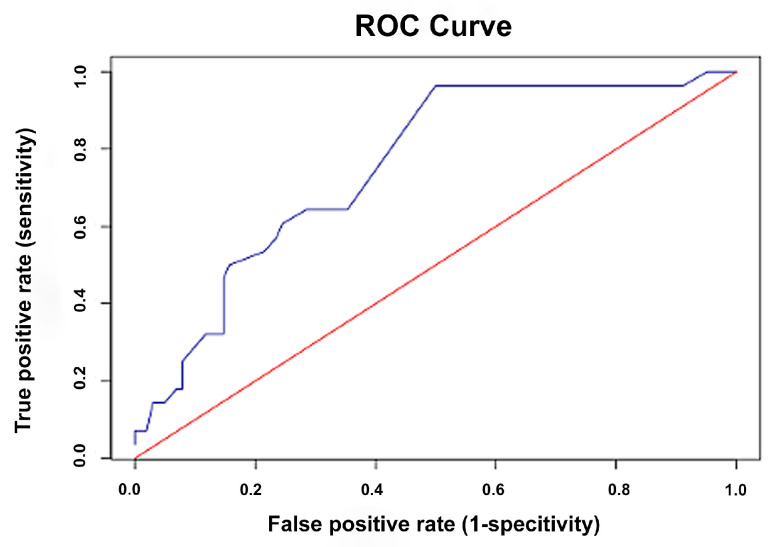
Receiver operating characteristic (ROC) curve for the owner-assessed feline pain scale in detecting pain requiring intervention, as determined by the Colorado State University Feline Acute Pain Scale (CSU-FPS). The blue line represents the ROC curve for the owner-assessed feline pain scale, and the red diagonal line represents the line of no discrimination.

ROC curve analysis was performed to evaluate the diagnostic accuracy of the owner-assessed feline pain scale in identifying cats requiring analgesic intervention as determined by established veterinary pain scales. The areas under the curve (AUC) for the owner-assessed scale were 0.87 (95% CI: 0.81–0.94) for the CMPS-Feline (Figure 2), 0.79 (95% CI: 0.71–0.87) for the FGS (Figure 3), and 0.75 (95% CI: 0.66–0.85) for the CSU-FPS (Figure 4), indicating good to excellent discriminatory performance. The following guidelines apply for interpreting the AUC: 0.5 = no discrimination; 0.7–0.8 = acceptable discrimination; 0.8–0.9 = excellent discrimination; ≥0.9 = outstanding discrimination [28]. Based on Youden’s J statistic, the optimal cut-off score for the owner-assessed scale was identified as 9 points. At this threshold, the sensitivity and specificity for detecting pain that warranted analgesic intervention were 96% and 78% using the CMPS-Feline, 95% and 57% using the FGS, and 96% and 50% using the CSU-FPS, respectively.

## 4. Discussion

This study developed and validated a behavior-based, owner-assessed feline acute pain scale, which demonstrated strong psychometric properties in a diverse clinical population. Key findings included the strong to moderate positive correlation of the scale with veterinary assessments using the CMPS-Feline, FGS, and CSU-FPS; substantial agreement with the CMPS-Feline; and the ability to differentiate levels of pain in cats that were consistent with the veterinary tools. Regarding reliability, the scale demonstrated acceptable internal consistency. These results suggest that the newly developed owner-assessed scale’s capability in capturing pain signs in cats aligns well with that of the validated professional veterinary assessments.

Pain assessment in cats remains a considerable challenge due to their tendency to mask clinical signs and the often subtle or ambiguous nature of pain-related behaviors [1,6,7,8]. This difficulty may be amplified in nonclinical settings, where owners or untrained carers may struggle to detect minor or context-dependent signs of pain [29,30]. While existing veterinary pain assessment tools, such as the CMPS-Feline and FGS, are available, their reliance on trained observation and variability in agreement among different groups of assessors [16,31,32,33,34,35] may limit their utility for owners without formal training. In contrast, the scale developed in this study was specifically designed for use by general cat owners without additional training, focusing on overt and commonly observable behaviors in the home environment. It was found that when provided with such a structured and validated tool, owners can effectively assess acute pain in their cats, potentially enhancing pain recognition outside clinical settings.

The development of an effective health measurement scale should incorporate several fundamental principles, such as validity, reliability, and feasibility [36]. The construct validity—defined as how well a tool measures the unobservable theoretical concept it intends to quantify [37]—of the owner-assessed feline pain scale was evidenced by its ability to differentiate pain levels between groups of cats. As shown by veterinary pain assessments, significant differences in pain scores were observed among the orthopedic, soft tissue, and postoperative groups (*p* < 0.001 for all veterinary scales). Notably, the owner-assessed scale also demonstrated statistically significant differences across these groups (*p* = 0.015), effectively capturing the expected variation in pain levels. Higher pain was observed in fracture cases (orthopedic group) compared to lower pain in postoperative cats, consistent with veterinary assessments. This ability to discriminate between different pain intensities in a clinical context provides strong support for the scale’s construct validity.

Strong correlations with established veterinary scales are pivotal for the clinical applicability of the owner-assessed scale. These correlations demonstrated the criterion validity—which refers to evidence of a relationship between the attributes in a measurement tool with its performance on some other variable [37]—of the owner-assessed scale when compared to the veterinary pain assessment tools. A strong positive correlation was found with the CMPS-Feline, while moderate positive correlations were observed with the CSU-FPS and FGS. This indicates that owner observations, when guided by the structured tool, largely correspond with professional veterinary assessments. The slightly stronger correlation with the CMPS-Feline (rho = 0.66) and CSU-FPS (rho = 0.57), compared to the FGS (rho = 0.53), may be attributed to the scale’s primary focus on overt behavioral indicators, such as changes in activity, posture, and vocalization. These indicators are also central components of the CMPS-Feline and CSU-FPS, while the FGS primarily assesses more subtle facial expressions. Nevertheless, the moderate correlation and agreement with the FGS were still valuable, indicating consistency across different observational domains. The substantial agreement (kappa = 0.74) with the CMPS-Feline is particularly encouraging, as it reflects a high degree of concordance between owner and veterinary conclusions, highlighting the potential for owner-derived information to be highly valuable in a clinical context. This finding is significant because the CMPS-Feline is a well-validated and widely used pain scale in veterinary practice.

It is worth noting that a deliberate design choice for our scale was not to prioritize subtle facial expressions, such as whiskers and muzzle tension, as primary components. Although previous research has demonstrated that cat owners can assess pain with good reliability using the FGS, it involved raters who had received prior training [15], and the tool was tested using static photographs, which may not reflect the dynamic nature of real-time pain assessment. The use of facial expressions may also be less feasible in certain cats, particularly brachycephalic (flat-faced) breeds or those with facial deformities. Therefore, by focusing on overt and observable behaviors, the scale developed in this study provides a more accessible tool for initial pain assessments in nonclinical settings, allowing for effective evaluation in a broader range of cats where facial cues cannot be reliably used.

The owner-assessed feline pain scale demonstrated good internal consistency reliability, with a standardized Cronbach’s alpha coefficient of 0.76, which exceeds the acceptable threshold of 0.70 for new scales [37]. Internal consistency measures how well the individual items of a tool correspond to the overall concept [37], and this finding suggests an adequate level of cohesiveness among the 13 items, indicating they consistently measure the underlying construct of feline pain. The recalculated coefficients when deleting individual items all ranged from 0.72 to 0.76, further confirming that all items contributed appropriately to the overall consistency, with none warranting removal. While acknowledging that Cronbach’s alpha tends to increase with longer scales [36] and considering the impact of sample size on this coefficient, our 13-item scale with a sample size of 130 yielded an alpha of 0.76, which falls within the ‘Fair’ to ‘Moderate’ range according to guidelines that factor in both scale length (≥12 items) and sample size (100–300 participants) [26].

In addition, the owner-assessed scale also demonstrated promising diagnostic accuracy in identifying cats requiring analgesic intervention as determined by established veterinary scales. The AUC values indicated good discriminatory performance when measured against the CMPS-Feline (0.87), with acceptable discrimination when compared to the FGS (0.79) and CSU-FPS (0.75) [38]. The optimal cut-off score of 9 points provides a practical threshold for owners. At this threshold, the scale exhibited high sensitivity (95–96%) for detecting pain that warrants analgesic intervention, although specificity varied across scales (78% for the CMPS-Feline, 57% for the FGS, and 50% for the CSU-FPS). High sensitivity is particularly valuable in pain assessment, as failing to identify cats in pain could result in undertreatment and adverse welfare outcomes. This cut-off score can empower owners to make informed decisions about seeking veterinary care, potentially leading to earlier intervention and better pain management. However, caution should be exercised, as this threshold should not be interpreted as a mandate to delay pain management if the score is below 9, nor as a substitute for professional veterinary consultation. Instead, it should serve as a guide for owners to monitor their cats closely and consult a veterinarian if they are uncertain.

The feasibility of the owner-assessed scale was promising for clinical applications. A key finding supporting this was the high rate of independent completion by owners, with only a small subset of elderly participants (n = 5) requiring assistance, indicating the scale’s ease of use across a wide age range (13 to 84 years). This suggests that the tool is user-friendly and minimally burdensome for the majority of cat owners, which is crucial for its potential adoption in home settings. The successful recruitment of a diverse sample of 130 cats across various trauma, disease, and postoperative conditions, along with a broad demographic of owners, further highlights the practicality of implementing this scale in routine practice. Additionally, the scale’s high sensitivity and acceptable specificity for identifying pain requiring analgesic intervention support its utility as a reliable screening or monitoring tool for owners.

The development of owner-assessed feline pain scales is not a novel concept, and several validated instruments have emerged over the past decade, demonstrating their reliability and utility in various contexts. Notable examples include the feline musculoskeletal pain index [38], the Montreal instrument for cat arthritis testing for use by caretakers [19], and client-specific outcome measures [18]. These scales have consistently shown good psychometric properties, such as validity and reliability, enabling owners to effectively monitor pain in their cats. However, these scales were specifically designed and validated for the assessment of pain associated with osteoarthritis (OA). Consequently, their item content predominantly focuses on subtle, long-term changes in behavior related to mobility, daily activities (e.g., jumping, grooming, litter box use), and social interaction, which are characteristic indicators of chronic musculoskeletal discomfort. While invaluable for longitudinal monitoring of conditions like OA, these scales may not fully capture the distinct behavioral manifestations of acute pain.

On the other hand, this current study specifically developed and validated an owner-assessed scale for use in cats experiencing acute pain, encompassing a diverse range of conditions. This distinction is crucial as it addresses a current gap in owner-assessed tools that are tailored for acute pain assessment. The newly developed owner-assessed scale provided robust evidence for its criterion validity when compared to established veterinary acute pain scales (the CMPS-Feline, FGS, CSU-FPS), which are widely recognized for their utility in acute pain scenarios. This potentially enables owners to provide valuable, real-time insights into acute pain, thereby facilitating more timely and appropriate veterinary intervention for their cats in the home environment, where signs of acute pain might otherwise go unnoticed or misinterpreted by owners relying solely on their intuition.

Of note, the cats included in this study were recruited from a surgical consultation setting and represented a range of acute pain conditions—including those awaiting orthopedic or soft tissue procedures and those undergoing postoperative states. While some of these cases involved surgical pain, others may have experienced trauma-related or disease-associated pain prior to surgery. This selection strategy supports the scale’s relevance for identifying acute pain in a clinical context where painful conditions are likely, though not exclusively surgical in origin. Future studies should aim to validate the tool in a broader population, including cats with a greater variety of pain types and severities, to better assess its generalizability across diverse pain presentations.

Another notable finding is that the strength of the owner-cat relationship did not significantly influence the pain scores assigned by the owners (rho = −0.15, *p* = 0.09). This result is important, as it addresses a potential concern regarding the objectivity of owner assessments. Previous research has explored the influence of owner-related factors, such as empathy and attitudes toward pets, on the perception of animal pain. For instance, Ellingsen et al. (2010) found that empathy was a predictor of how people rated pain in dogs [39]. In contrast to those findings, the present study indicates that the strength of the owner-cat relationship did not significantly influence pain scores. This might imply that even highly bonded owners did not tend to overestimate their cats’ pain, and those with less interaction were still able to recognize relevant pain-related behaviors. It is important to note, however, that the majority of cat owners in this study reported strong relationships with their pets; therefore, further research should include a more diverse population to confirm these findings.

Similarly, the age and gender of owners were not associated with the pain scores derived from the new scale. This finding is noteworthy, as some previous studies have indicated that gender could influence pain assessment, with females potentially rating pain scores higher [40,41]. The lack of such an association in the present study further supports the objectivity and usability of the owner-assessed scale across varying levels of owner-cat familiarity and demographic characteristics. It suggests that the structured nature of the scale may mitigate the influence of individual owner attributes, thereby enhancing generalizability and reducing potential bias in pain assessment across diverse household environments. Nonetheless, it should be noted that a small number of elderly participants required assistance to complete the scale, as mentioned above, which highlights a potential usability consideration in older age groups.

Pain in animals is widely recognized as a multidimensional experience that encompasses physiological, behavioral, and emotional components [42]. The present tool was intentionally designed to focus on behavioral manifestations, which are the most accessible and reliable for owners to recognize in daily settings. Nonetheless, several items in the scale, such as hiding, decreased social interaction, and increased aggression, may also indirectly reflect the emotional distress associated with pain. Although the physiological and emotional dimensions were beyond the scope of this study, their relevance underscores the complexity of pain assessment and points to the potential value of incorporating these aspects in refining owner-based tools in future research.

Despite these promising results, this study has several limitations. Firstly, the assessment of test–retest reliability was not feasible due to ethical considerations necessitating immediate analgesic administration post-assessment, which would alter the cats’ pain states. Similarly, inter-rater reliability could not be directly assessed. This was primarily due to most owners bringing their cats for treatment alone, making it challenging to include multiple caregivers for a single cat within the study’s scope. Future studies could explore inter-rater reliability by specifically selecting cases where more than one owner or caregiver from the same household brings the cat to the hospital. This approach would allow for the quantification of assessment consistency between different individuals within the same household or across various owners, providing a more comprehensive understanding of how consistent pain scores are when multiple people are involved in the cat’s care. Secondly, while the study included cats with various acute pain conditions (traumatic, disease-associated, and surgical), the generalizability of these findings needs further evaluation across a broader range of pain etiologies and severities. Thirdly, owner assessments were performed in the hospital setting based on home observations, which could introduce recall bias, particularly if owners had difficulty recalling specific behaviors. Future studies incorporating repeated home-based assessments will be valuable to confirm the tool’s applicability in real-life settings. Fourthly, cats presenting for elective procedures were excluded, although such cases could have served as a negative control group not expected to experience pain. Including these cases would have further established the discriminant validity of the tool by confirming that owners do not assign pain scores to apparently healthy cats. Future studies incorporating a negative control group will be valuable to refine the proposed cut-off score and further strengthen the clinical applicability of this tool. Additionally, unassessed owner-related factors, such as prior experience with cats or general observational skills, may have influenced pain detection accuracy, but these variables were not formally assessed. Lastly, although the scale was designed for simplicity, a small number of participants required assistance from the research team during the assessment process. This direct intervention from the researchers could have influenced the owners’ responses and potentially introduced a form of observer bias.

## 5. Conclusions

In conclusion, the newly developed owner-assessed feline acute pain scale represents a promising and practical tool for detecting acute pain in cats at home. Its robust psychometric properties, strong correlation with veterinary assessments, and high diagnostic accuracy, coupled with its ease of use, suggest substantial potential to enhance feline pain management. The scale empowers owners to recognize acute pain through structured behavioral criteria, enabling earlier recognition of pain and more timely decisions about seeking veterinary care. In addition, information derived from this tool may provide useful context for veterinarians when evaluating patients, further supporting comprehensive pain management. Continued refinement, real-world testing, and broader application across diverse pain types will be essential in advancing its clinical relevance and integrating it into routine veterinary practice. Future studies should also expand the sample size and diversity to ensure applicability across all acute pain presentations in cats.

## Figures and Tables

**Table 1:** Demographic characteristics of 130 cats by clinical groups.

Characteristic	Orthopedic (n = 48)	Soft Tissue (n = 36)	Postoperative (n = 46)	Total (n = 130)
Cat age (years), median (IQR)	1.0 (0.3, 2.8)	4.0 (2.3, 7.8)	4.0 (1.3, 6.1)	2.3 (0.8, 5.8)
Sex, n (%)				
Male	24 (50.0%)	23 (63.9%)	24 (52.2%)	71 (54.6%)
Female	24 (50.0%)	13 (36.1%)	22 (47.8%)	59 (45.4%)
Breed, n (%)				
Domestic Shorthair	40 (83.3%)	27 (75.0%)	34 (73.9%)	101 (77.7%)
Persian	2 (4.2%)	3 (8.3%)	5 (10.9%)	10 (7.7%)
Scottish Fold	4 (8.3%)	3 (8.3%)	3 (6.5%)	10 (7.7%)
American Shorthair	1 (2.1%)	1 (2.8%)	1 (2.2%)	3 (2.3%)
Devon Rex	0 (0.0%)	1 (2.8%)	0 (0.0%)	1 (0.8%)
British Longhair	1 (2.1%)	1 (2.8%)	2 (4.3%)	4 (3.1%)
Maine Coon	0 (0.0%)	0 (0.0%)	1 (2.2%)	1 (0.8%)

IQR = interquartile range.

**Table 2:** Pain scores of cats in orthopedic, soft tissue, and postoperative groups, assessed using the owner-assessed pain scale and three veterinary pain assessment tools: the Glasgow Composite Measure Pain Scale-Feline (CMPS-Feline), the Feline Grimace Scale (FGS), and the Colorado State University Feline Acute Pain Scale (CSU-FPS).

Group	Owner-Assessed Scale Median (IQR)	CMPS-Feline Median (IQR)	FGS Median (IQR)	CSU-FPS Median (IQR)
Orthopedic	9.0 (8.0, 16.0) ^a^	7.0 (3.0, 9.0) ^a^	3.0 (1.0, 5.0) ^a^	1.75 (0.75, 2.00) ^a^
Soft tissue	10.0 (5.0, 17.8) ^a,b^	5.0 (1.0, 7.0) ^b^	2.0 (0.0, 4.0) ^a,b^	0.75 (0.00, 1.69) ^b^
Postoperative	8.0 (3.0, 10.3) ^b^	3.0 (2.0, 6.0) ^b^	1.0 (0.0, 3.0) ^b^	0.50 (0.25, 1.00) ^b^
*p*-value	0.015	<0.001	<0.001	<0.001

Total score ranges: Owner-assessed scale 0–33; CMPS-Feline 0–20; FGS 0–10; CSU-FPS 0–4. Different superscript letters within each column indicate statistically significant differences (*p* < 0.05) in pain scores among groups for that specific pain scale. IQR = interquartile range.

**Table 3:** Spearman’s rank correlation between the owner-assessed pain assessment scale and veterinary pain tools, including the Glasgow Composite Measure Pain Scale-Feline (CMPS-Feline), Feline Grimace Scale (FGS), and Colorado State University Feline Acute Pain Scale (CSU-FPS).

Pain Scaling System	CMPS-FELINE Rho (95% CI)	FGS Rho (95% CI)	CSU-FPS Rho (95% CI)
Owner-assessed pain scale	0.66 (0.50–0.72)	0.53 (0.38–0.66)	0.57 (0.44–0.68)
CMPS-Feline	-	0.79 (0.70–0.85)	0.83 (0.76–0.89)
FGS	-	-	0.63 (0.51–0.73)

Interpretation of Spearman’s rank correlation (rho):  < 0.19  =  very weak; 0.20–0.39  =  weak; 0.4–0.59  =  moderate; 0.6–0.79  =  strong; 0. 8–1.0  =  very strong [14]. CI = confidence interval.

**Table 4:** Cohen’s kappa values assessing agreement between the owner-assessed pain assessment scale and veterinary pain tools, including the Glasgow Composite Measure Pain Scale-Feline (CMPS-Feline), Feline Grimace Scale (FGS), and Colorado State University Feline Acute Pain Scale (CSU-FPS).

Pain Scaling System	CMPS-Feline Kappa (95% CI)	FGS Kappa (95% CI)	CSU-FPS Kappa (95% CI)
Owner-assessed pain scale	0.74 (0.57–0.91)	0.44 (0.23–0.59)	0.28 (0.16–0.41)
CMPS-Feline	-	0.57 (0.43–0.71)	0.38 (0.22–0.54)
FGS	-	-	0.37 (0.19–0.56)

Cohen’s kappa values interpret the level of agreement as follows: <0.00 = poor agreement, 0.00–0.20 = slight agreement, 0.21–0.40 = fair agreement, 0.41–0.60 = moderate agreement, 0.61–0.80 = substantial agreement, and 0.81–1.00 = almost perfect agreement [26]. CI = confidence interval.

## Data Availability

The original contributions presented in this study are included in the article/Supplementary Materials. Further inquiries can be directed to the corresponding author.

## Supplementary Materials

The following supporting information can be downloaded at: https://www.mdpi.com/article/10.3390/ani15192801/s1, Table S1: The dataset of study population and pain assessment scores.

## Abbreviations

The following abbreviations are used in this manuscript:

CMPS-Feline	Glasgow Composite Measure Pain Scale-Feline
FGS	Feline Grimace Scale
CSU-FPS	Colorado State University Feline Acute Pain Scale
MCP	Multidimensional Composite Pain Scale
TECA	Total Ear Canal Ablation
CVI	Content Validity Index
VAS	Visual Analog Scale
ROC	Receiver Operating Characteristic
IQR	Interquartile Range
CI	Confidence Interval
AUC	Area Under the Curve
OA	Osteoarthritis
